# Inhibition of Notch Signaling by a γ-Secretase Inhibitor Attenuates Hepatic Fibrosis in Rats

**DOI:** 10.1371/journal.pone.0046512

**Published:** 2012-10-03

**Authors:** Yixiong Chen, Shaoping Zheng, Dan Qi, Shaojiang Zheng, Junli Guo, Shuling Zhang, Zhihong Weng

**Affiliations:** 1 Department of Infectious Disease, Union Hospital, Tongji Medical College, Huazhong University of Science & Technology, Wuhan, People’s Republic of China; 2 Department of Ultrasonography, Union Hospital, Tongji Medical College, Huazhong University of Science & Technology, Wuhan, People’s Republic of China; 3 Department of Pathology and Hainan Provincial Key Laboratory of Tropical Medicine, Hainan Medical College, Haikou, People’s Republic of China; University of Valencia, Spain

## Abstract

Notch signaling is essential to the regulation of cell differentiation, and aberrant activation of this pathway is implicated in human fibrotic diseases, such as pulmonary, renal, and peritoneal fibrosis. However, the role of Notch signaling in hepatic fibrosis has not been fully investigated. In the present study, we show Notch signaling to be highly activated in a rat model of liver fibrosis induced by carbon tetrachloride (CCl_4_), as indicated by increased expression of Jagged1, Notch3, and Hes1. Blocking Notch signaling activation by a γ-secretase inhibitor, DAPT, significantly attenuated liver fibrosis and decreased the expression of snail, vimentin, and TGF-β1 in association with the enhanced expression of E-cadherin. The study *in vitro* revealed that DAPT treatment could suppress the EMT process of rat hepatic stellate cell line (HSC-T6). Interestingly, DAPT treatment was found not to affect hepatocyte proliferation *in vivo*. In contrast, DAPT can inhibit hepatocyte apoptosis to some degree. Our study provides the first evidence that Notch signaling is implicated in hepatic fibrogenesis and DAPT treatment has a protective effect on hepatocytes and ameliorates liver fibrosis. These findings suggest that the inhibition of Notch signaling might present a novel therapeutic approach for hepatic fibrosis.

## Introduction

Hepatic fibrosis is a reversible wound-healing response characterized by the accumulation of extracellular matrix (ECM) in response to acute or chronic liver injury. Perpetuation of the fibrotic reaction can lead to end-stage liver disease, cirrhosis, and hepatocellular carcinoma, whose incidence is increasing worldwide [1]. The activation and proliferation of hepatic stellate cells (HSCs) has been identified as a critical event in the development of hepatic fibrosis. Activated HSCs are highly contractile and express α-smooth muscle actin (α-SMA) and ECM. They are a key target for anti-fibrotic therapies because these cells are the primary source of ECM in injured livers [2]–[5].

The Notch signaling pathway is a highly conserved signal transduction mechanism. It is essential to normal embryonic development, cellular proliferation, specification, and differentiation [6]. Four Notch receptors (Notch1, Notch2, Notch3, and Notch4) and five ligands (Jagged1, Jagged2, Delta1, Delta3, and Delta-like 4) have been identified in mammals [7]–[15]. Notch signaling is activated through an interaction of a Notch receptor with a ligand expressed on adjacent cells leading to proteolytic cleavages of Notch receptor. The cleavage step catalyzed by the γ-secretase complex results in the release of the Notch intracellular domain (NICD) [16]. The NICD then moves to the nucleus, where it interacts with CSL (RBP-Jk/CBF1) and Mastermind to activate transcription of downstream target genes such as Hes1 (hairy and enhancer of split 1), HRT (hairy-related transcription), Deltes-1, Meltrin-β, and the Notch receptors themselves [17]–[20].

Notch signaling is essential to the regulation of cell differentiation, and aberrant activation of this pathway is implicated in the pathogenesis of several malignancies [21]–[22]. Increasing numbers of studies have reported that Notch signaling is involved in human fibrotic diseases [23]–[25]. However, the role of Notch signaling in liver fibrosis has not been fully investigated. Previous studies have indicated that all 4 receptors are expressed in the adult liver, with no significant differences in the levels of Notch1, 2, and 4 mRNA between normal and diseased livers [26]. However, the expression of Notch3 and Jagged1 protein has been found to be significantly up-regulated in diseased liver tissue [26]–[27]. Recent research has found the mRNA of Notch receptors (Notch1, 2, and 4) to be present in freshly isolated rat HSCs, which displayed no protein synthesis of Notch ligands (Jagged1 and Jagged2). However, the amount of Jagged1 protein increased while isolated HSCs developed into myofibroblast-like cells [28]. Based on these studies, we hypothesize that Notch signaling might be involved in liver fibrogenesis.

In the present study, we investigated the role of Notch signaling during the process of liver fibrosis and clarified its mechanism. Our results demonstrated that Notch signaling is activated in hepatic fibrosis induced by CCl_4_ and that blocking Notch signaling using γ-secretase inhibitor can significantly attenuate liver fibrosis. These results suggest that selective interruption of Notch signaling might be a novel anti-fibrotic strategy in hepatic fibrosis.

## Results

### 1. Activation of Notch Signaling in a Rat Model of Liver Fibrosis Induced by CCl_4_

**Figure 1.Transcripts:**
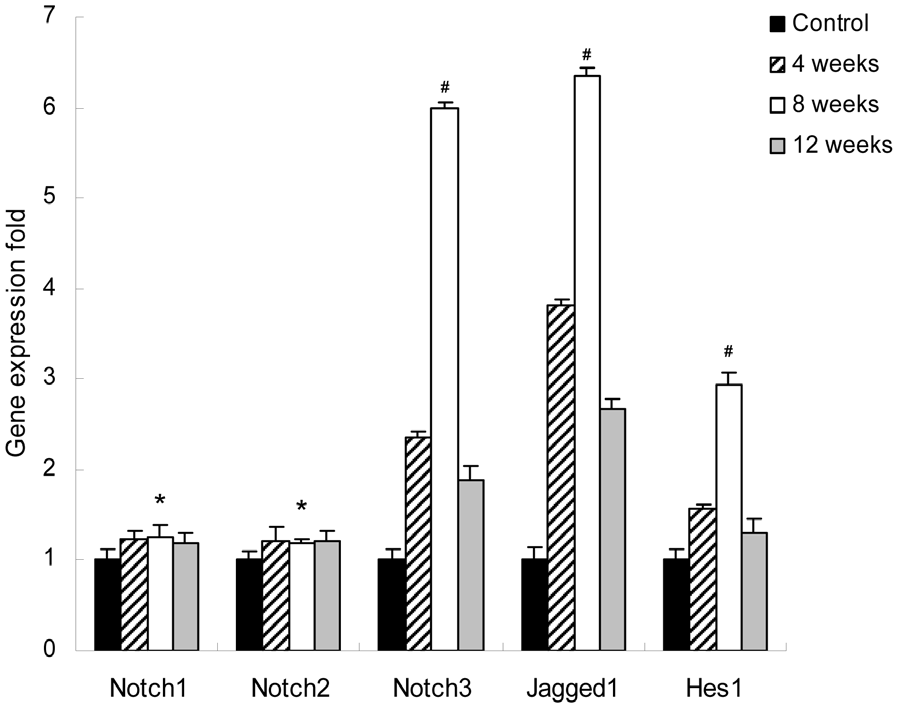
of Notch signaling components in fibrotic liver. Real-time PCR was performed to detect mRNA levels of Notch1, Notch2, Notch3, Jagged1, and Hes1 in rats treated with olive oil or CCl_4_. The mRNA expression levels were normalized against GAPDH. Gene expression folds in model group were normalized by that of normal group. Each value represents the mean for triplicate samples. *P>0.05 versus rats at 4 weeks or 12 weeks. ^#^ *P*<0.05 versus rats at 4 weeks or 12 weeks.

**Figure 2:**
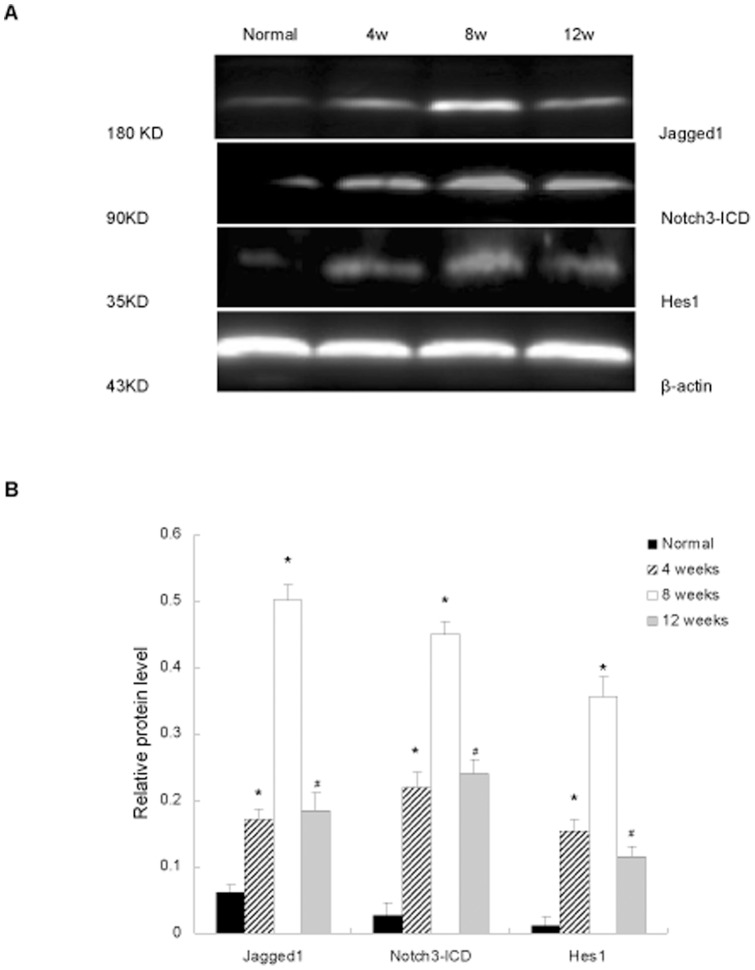
The protein levels of Notch signaling components in fibrotic liver. **A**. Rats treated with olive oil or CCl_4_ were killed. The protein levels of Notch3-ICD, Jagged1, and Hes1 were analyzed by Western blot. **B.** The expression was normalized against β-actin. *P<0.05 versus rats in normal group. ^#^ *P*<0.05 versus rats at 8 weeks.

To investigate the expression of Notch signaling components during liver fibrosis, rats were treated with CCl_4_ and killed as described in Materials and Methods. As shown in Figure 1, the mRNA levels of Jagged1, Notch3, and Hes1 was gradually increased after CCl_4_ treatment. Significantly higher levels were detected in the 8 week group, but expression decreased after the termination of CCl_4_ injection. The mRNA levels of Notch1 and Notch2 showed no markedly change during liver fibrogenesis. The protein levels of Jagged1, Notch3-ICD (intracellular domain), and Hes1 showed the same changes in accordance with their mRNA levels during liver fibrogenesis by Western blot analysis (Figure 2). The levels of expression of Notch1-ICD and Notch2-ICD proteins did not change significantly during liver fibrosis, as indicated by Western blot analysis. This is consistent with the stability of the corresponding mRNA levels (Figure S1).

**Figure 3:**
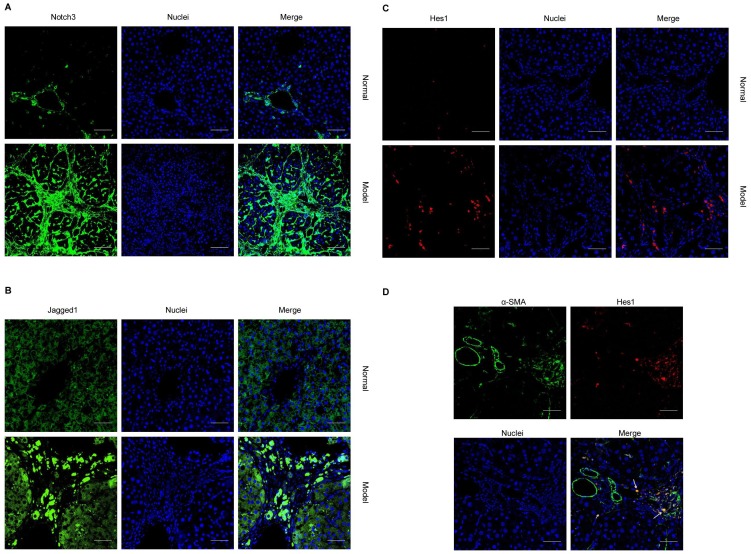
Immunofluorescence evidence for increased Notch signaling activation in fibrotic livers. Liver sections of rats after 8 weeks of CCl_4_ or olive oil treatment were stained with antibodies against Notch3, Jagged1, Hes1, and α-SMA. **A.** Notch3. **B.** Jagged1. **C.** Hes1. **D.** Liver sections of rats after 8 weeks of CCl_4_ treatment were costained with antibodies against Hes1 (red) and α-SMA (green). Nuclei were stained with DAPI (blue). Images were taken by confocal fluorescent microscopy and the white bars represent 50 µm. Arrows indicate Hes1/α-SMA double positive cells.

The expression of Notch3, Jagged1, and Hes1 was confirmed by immunofluorescence. As shown in Figure 3, positive staining for Notch3 and Hes1 was significantly enhanced in the activated HSCs in fibrotic livers of the rats treated with 8 weeks of CCl_4_. In contrast, few cells with positive staining for Notch3 and Hes1 expression were detected in liver tissues from normal group. Weak Jagged1 staining for hepatocytes was seen in livers of the rats in normal group, while the expression of Jagged1 was dramatically increased in fibrotic livers of the rats treated with CCl_4_. Interestingly, some hepatocytes close to the fibrotic area expressed high levels of Jagged1. In fibrotic livers, Hes1–positive staining was co-expressed with α-SMA (Figure 3D). These data indicate that the up-regulation of Notch signaling is activated in fibrotic livers and participates in the activation of HSCs.

### 2. DAPT Attenuates CCl_4_-induced Liver Fibrosis in Rats

**Figure 4:**
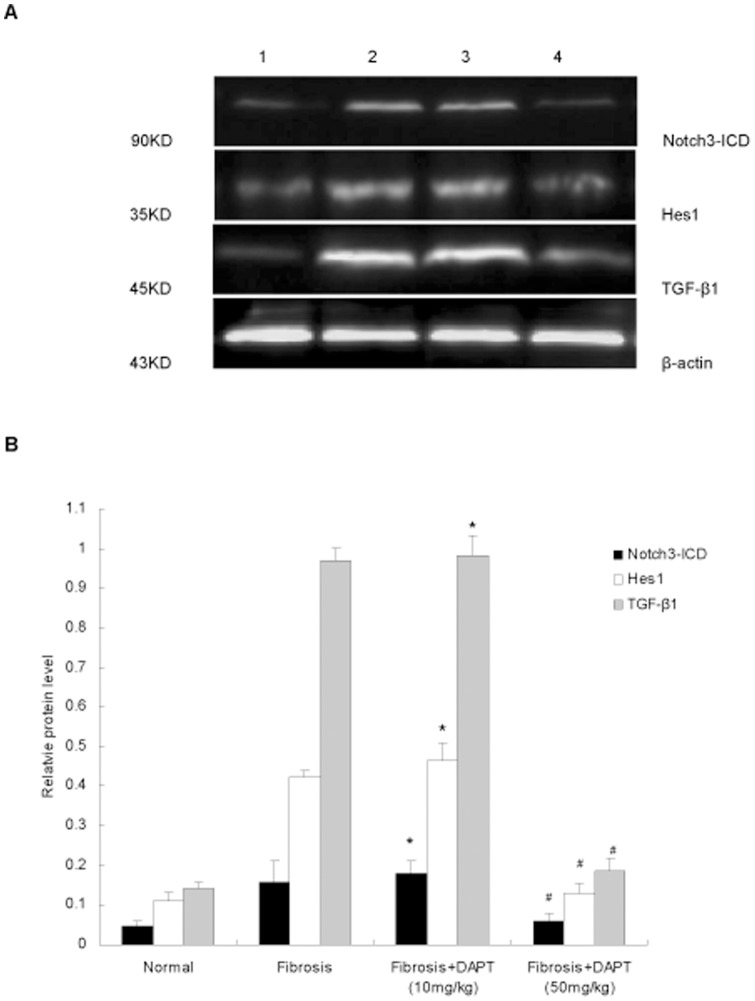
Effects of DAPT on inhibiting Notch signaling activation in fibrotic liver. A. The protein levels of Notch3-ICD, Hes1, and TGF-β1 were examined by Western blot. **B.** The expression was normalized against β-actin. *P>0.05 versus rats in fibrosis group. ^#^ *P*<0.05 versus rats in fibrosis group. 1 indicates normal rats; 2 indicates rats in fibrosis group treated with DMSO and CCl_4_; 3 indicates rats treated with CCl_4_ and DAPT (10 mg/kg); 4 indicates rats treated with CCl_4_ and DAPT (50 mg/kg).

To further investigate whether blocking Notch signaling by a γ-secretase inhibitor DAPT could attenuate hepatic fibrosis *in vivo*. Rats were treated with DAPT as described in Materials and Methods. The 50 mg/kg dose of DAPT significantly reduced the protein levels of Notch3-ICD and Hes1 as compared with fibrosis group (*P*<0.05, Figure 4). But the 10 mg/kg dose had no significant effect (*P*>0.05, Figure 4).

**Figure 5:**
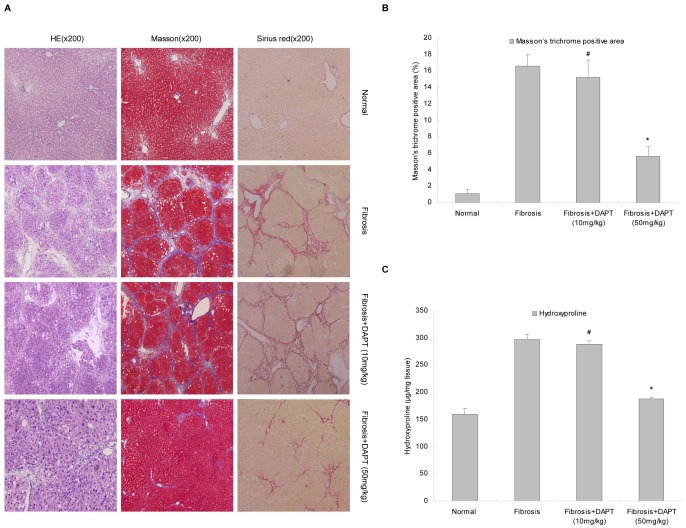
DAPT ameliorated liver fibrosis induced by CCl_4_ in rats. A. Hematoxylin and eosin, Masson’s trichrome, and Sirius red staining were used to examine pathological alterations and collagen deposition. Data shown are the representative of 8 animals. **B.** Semiquantitative analysis of the Masson’s trichrome staining result. **C.** Assay of hydroxyproline content. *P<0.05 versus rats in fibrosis group; ^#^ *P*>0.05 versus rats in fibrosis group.

DAPT (50 mg/kg) ameliorated the development of hepatic fibrosis, as confirmed by hematoxylin and eosin, Masson’s trichrome, and Sirius red staining (Figure 5A). The ECM area (Masson’s staining) was reduced by approximately 66.3% (*P*<0.05) as compared with fibrosis group (Figure 5B). The 10 mg/kg dose of DAPT had no significant anti-fibrotic effect (*P*>0.05, Figure 5B).

Quantitative analysis indicated less hydroxyproline content in the DAPT (50 mg/kg) treated group (187.63±3.30 µg/mg) than in the fibrosis group (297.38±9.42 µg/mg, *P*<0.05). There was no significant difference of the hydroxyproline content in DAPT (10 mg/kg) treated group (289.12±6.1 µg/mg) compared with that in the fibrosis group (*P*>0.05, Figure 5C). The hydroxyproline content in the normal group was 160.09±10.11 µg/mg in liver tissue (Figure 5C).

### 3. DAPT Attenuates Experimental Hepatic Fibrosis Through Inhibiting EMT

**Figure 6:**
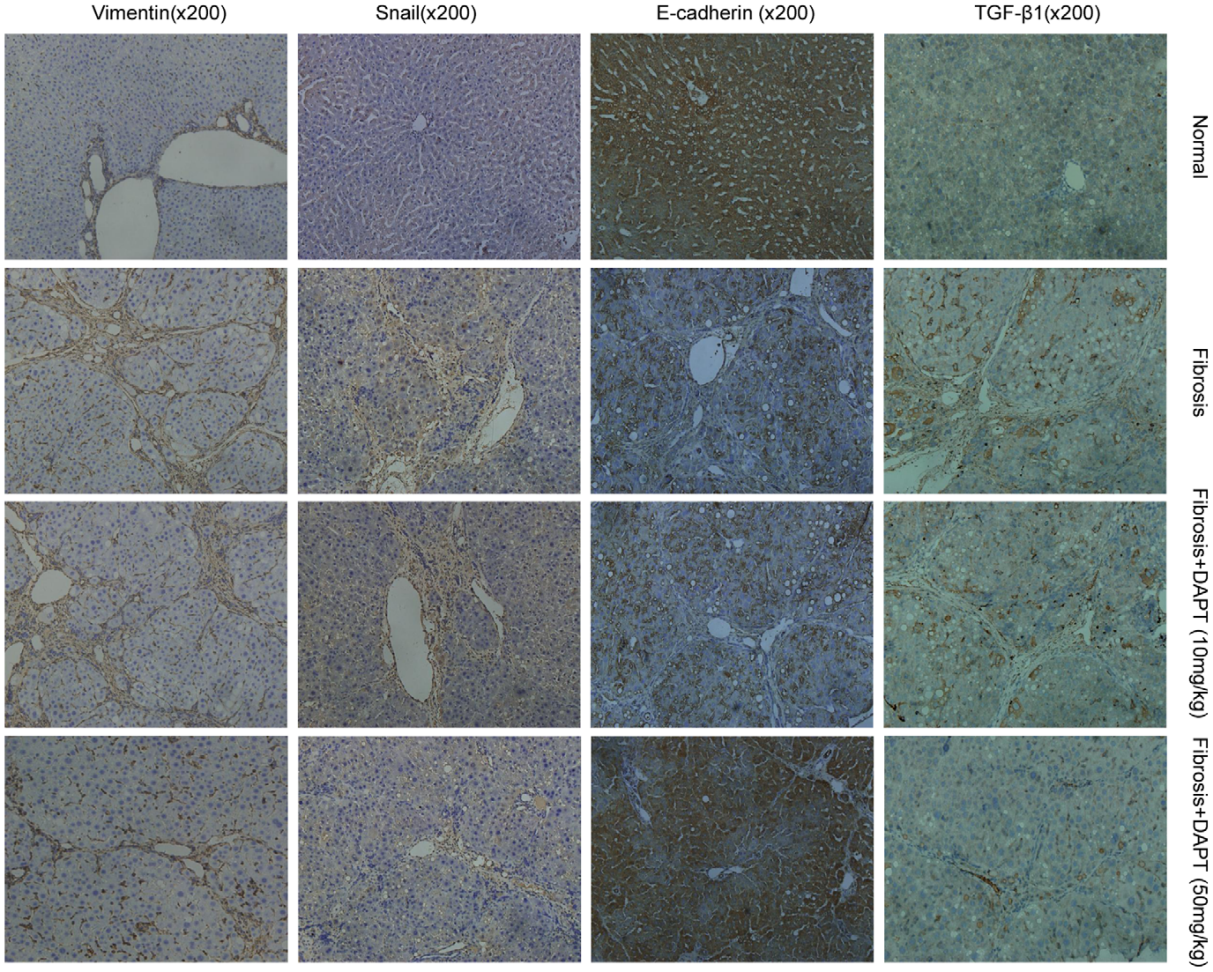
DAPT treatment was found to inhibit EMT in fibrotic livers in rats. Immunohistochemical staining was performed to detect the expression of vimentin, snail, E-cadherin, and TGF-β1 in livers from normal, fibrosis, and DAPT-treated rats.

We then analyzed the levels of EMT markers by immunohistochemical analysis. The expression of vimentin, snail, and TGF-β1 were markedly attenuated in DAPT (50 mg/kg) treated rats relative to the fibrosis group, and E-cadherin expression was increased significantly in DAPT (50 mg/kg) treated rats (Figure 6).

### 4. Effects of DAPT Treatment on Cell Proliferation and Apoptosis

**Figure 7:**
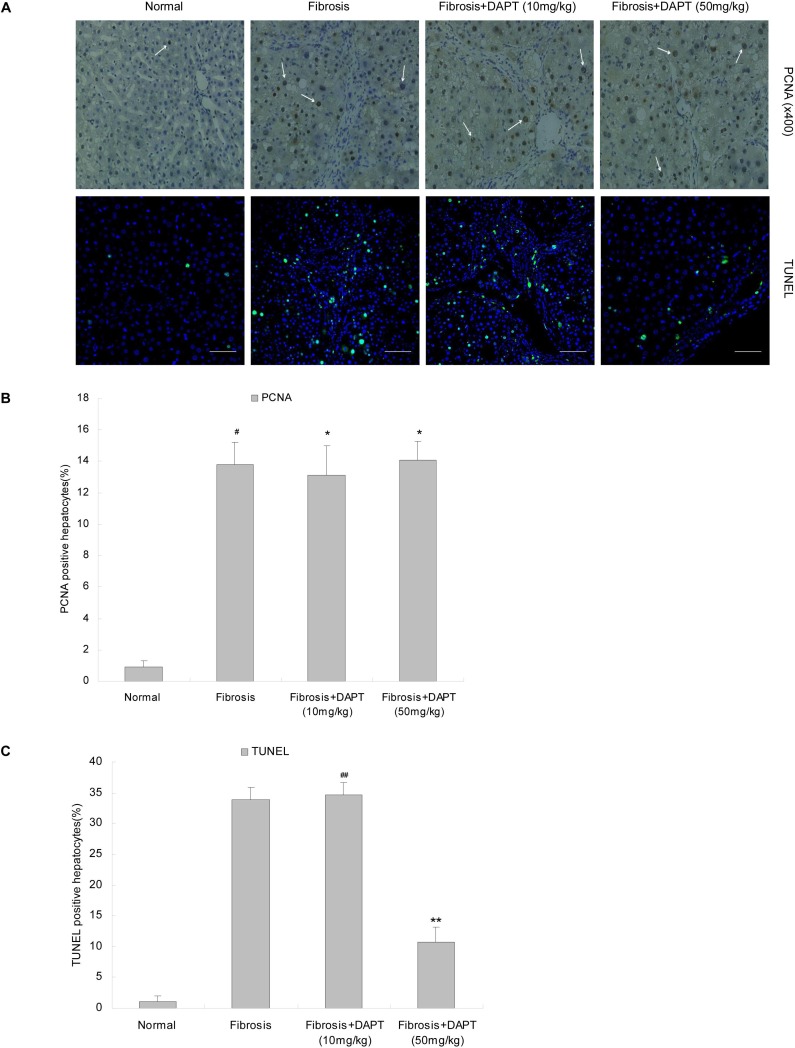
Effects of DAPT treatment on cell proliferation and apoptosis. **A.** Immunohistochemical and immunofluorescence staining were used to determine the expression of PCNA and TUNEL in fibrosis induced by CCl_4_ in liver tissues from normal, fibrosis, and DAPT-treated rats. **B, C.** Regions with positive PCNA or TUNEL staining were quantified using ImageJ as described in Materials and Methods. The white bars represent 50 µm. ^#^ *P*>0.05 versus DAPT-treated rats; *P<0.01 versus normal rats; ^##^ *P*>0.05 versus rats in fibrosis group; **P<0.05 versus rats in fibrosis group.

PCNA immunohistochemical staining was performed to determine whether DAPT treatment could affect cellular proliferation *in vivo*. The PCNA indices of hepatocytes treated with DAPT were higher than those of the normal group (*p*<0.01, Figure 7B). Moreover, there are no significant difference in hepatocyte proliferation in rats treated with DAPT compared with that of fibrosis group (*p*>0.05, Figure 7B).

TUNEL staining results revealed that the fibrotic liver specimens contained many apoptotic hepatocytes. However, DAPT (50 mg/kg) treatment had a strong protective effect on the hepatocytes against apoptosis (*p*<0.05, Figure 7C). The 10 mg/kg dose of DAPT had no significant protect effect (*P*>0.05, Figure 7C). The caspase 3 activity in liver lysates from normal, fibrosis, and DAPT-treated rats was detected by Western blot analysis. The results showed that the levels of cleaved caspase 3 increased significantly in fibrosis group and in rats treated with DAPT at a dose of 10 mg/kg, and it decreased markedly in rats given DAPT at a dose of 50 mg/kg (*p*<0.05, Figure S2). This confirms that DAPT (50 mg/kg) can inhibit hepatocyte apoptosis *in vivo.*

### 5. DAPT Treatment Inhibits EMT in HSC-T6 Cells

**Figure 8:**
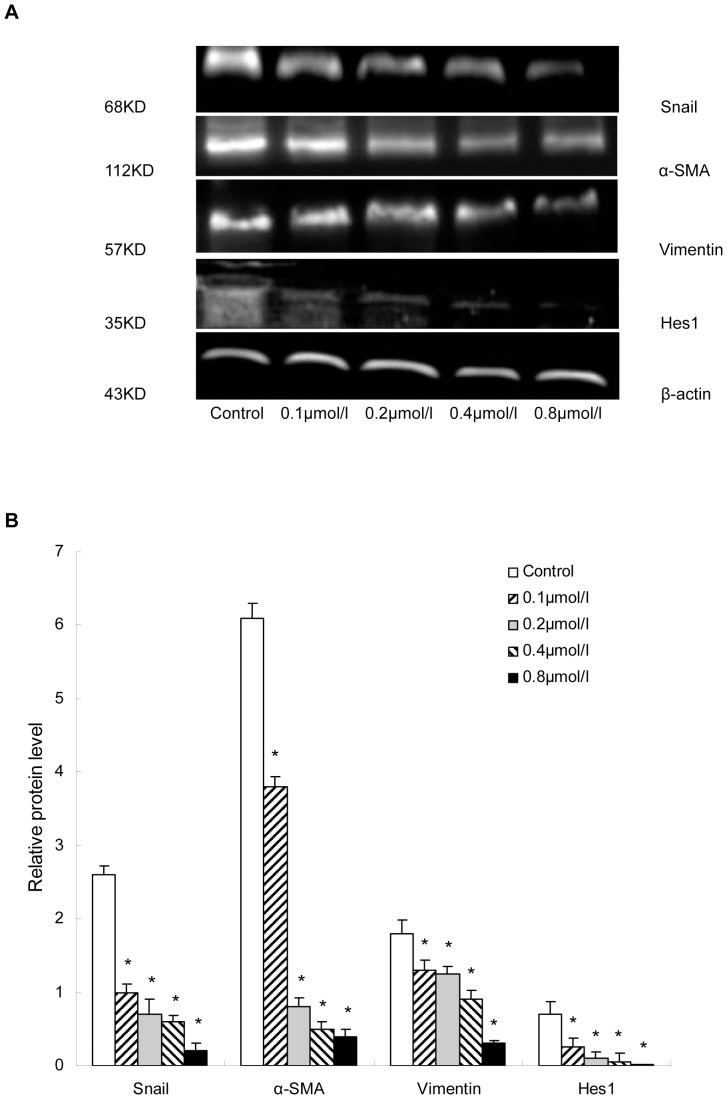
DAPT treatment inhibited EMT in HSC-T6 cells. Expression of snail, vimentin, Hes1, and α-SMA in HSC-T6 cells cultured with DAPT (0.1, 0.2, 0.4, and 0.8 µmol/l) or DMSO (control) for 48 h were analyzed by Western blot analysis. The expression was normalized against β-actin. *P<0.05 versus control group.

Next we investigated whether inhibition of the Notch pathway by DAPT could suppress the EMT in HSC-T6 cells. We first performed a Cell Counting Kit-8 assay to assess whether DAPT affected cell proliferation. No changes in metabolic activity were observed in cells exposed to DAPT at 0.5, 1, 2, 4, and 8 µmol/l (data not shown). We then treated HSC-T6 with DAPT to block Notch signaling. Expressions of snail, vimentin, Hes1, and α-SMA in HSC-T6 cultured for 48 h with DAPT or DMSO as a control were detected by Western blot analysis (Figure 8). The results showed that treatment with DAPT effectively reduced the expression of snail, vimentin, and Hes1 accompanied by α-SMA in HSC-T6 cells.

### 6. Knockdown of Notch3 by siRNA Inhibits EMT in HSC-T6 Cells

To determine the role of Notch3 in the EMT process in HSC-T6 cells, siRNA was employed to specifically knockdown Notch3. Western blot analysis showed that the expression of snail, vimentin, α-SMA, and Notch3-ICD in HSC-T6 cells was down-regulated 72 h after siRNA transfection (*p*<0.05, Figure S3).

## Discussion

In this study, we show that Notch signaling is markedly activated in a rat model of liver fibrosis induced by CCl_4._ Importantly, treatment with the γ-secretase inhibitor DAPT strongly inhibited the activation of HSCs. DAPT treatment also significantly attenuated CCl_4_-induced hepatic fibrosis *in vivo* as demonstrated by the decreased ECM accumulation. DAPT treatment was found not to affect hepatocyte proliferation but rather to inhibit hepatocyte apoptosis to some degree *in vivo*.

Notch signaling is involved in cell proliferation, survival, apoptosis, and differentiation, all of which affect the development and function of many organs [29]–[31]. Recently, Bielesz et al. reported that active Notch signaling pathways in tubular epithelial cells is a critical regulator of tubulointerstitial fibrosis [24]. Another study by Zhu et al. has demonstrated that Notch signaling is highly activated in rats in peritoneal dialysis fluid-induced fibrotic peritoneum [25]. We have previously shown that Notch3 significantly up-regulated in fibrotic liver tissues of patients with hepatitis [32]. The present study reveals that in fibrotic rat liver, the mRNA levels of Notch3, Jagged1, and Hes1 were markedly increased during liver fibrogenesis, and then decreased along with the resolution of fibrosis. This dynamic change was confirmed by western blot analysis. The upregulated expression of Notch3 and Hes1 by activated HSCs and the increased synthesis of Jagged1 by neighboring hepatocytes and activated HSCs itself suggest that Notch signaling is activated in rats with liver fibrosis induced by CCl_4._

Epithelial-mesenchymal transition (EMT) is defined as a process whereby epithelial cells gradually lose their epithelial signatures while acquiring the characteristics of mesenchymal cells [33], [34]. Numerous reports have indicated a role for EMT in fibrosis [1]. In particular, recent advances have confirmed that myofibroblasts can be supplemented from cholangiocytes and hepatocytes by EMT during hepatic fibrosis [35]–[38]. The Notch signaling pathway was also found to contribute to EMT. In human breast epithelial cells, Jagged1–mediated activation of Notch signaling induces EMT through induction of Slug and subsequent repression of E-cadheirn [39]. TGF-β1 is considered the most powerful inducer of EMT [39], and it has been demonstrated that TGF-β1 mediates EMT by the induction of snail, a repressor of E-cadherin transcription. Our current study reveals that γ-secretase inhibitor DAPT treatment reduced the numbers of myofibroblast-like cells and simultaneously inhibited expression of snail, vimentin, and TGF-β1 in parallel with enhanced expression of E-cadherin in fibrotic liver. These finding suggests that the reversion of EMT contributes to the resolution of hepatic fibrosis.

It has also been suggested that the activation of HSCs might be considered an EMT phenomenon [40]. The elements of the Notch signaling pathway, including Jagged1, Notch1, Notch2, and Hes1, have been identified as TGF-β1-responsive genes in kidney epithelia [41]. Our previously study showed that activated Notch signaling was found in HSC-T6 cells, and that transient knockdown of Notch3 antagonized TGF-β1-induced expression of α-SMA and collagen I in HSC-T6 [32]. In this study, the results show that the up-regulation of Notch signaling is implicated in the activation of HSCs *in vivo*. Moreover, treatment with DAPT effectively reduces the expression of α-SMA, snail, and vimentin in HSC-T6 cells. These data suggest that DAPT attenuated hepatic fibrosis at least partially through the inhibition of the EMT process of HSCs activation. The knockdown of Notch3 using siRNA was found to have the same impact on EMT in HSC-T6 cells as that of DAPT. This confirms that the Notch signaling pathway is a key regulator of EMT in HSC-T6 cells.

The conventional strategy for the treatment of hepatic fibrosis involves reducing the activation and proliferation of HSCs and inducing apoptosis. However, it has been reported that activated HSCs promote liver development and regeneration [42]. This suggests that it may not be appropriate to simply target HSCs to treat liver fibrosis. In the process of liver fibrosis, stimulation of hepatocyte regeneration and inhibition of apoptosis is essential to treating hepatic fibrosis [43]. In the present study, DAPT treatment was found not to inhibit hepatocyte proliferation. In contrast, DAPT was found likely to inhibit hepatocyte apoptosis to some degree *in vivo*. We also found that the expression of TGF-β1 was upregulated in the fibrotic livers, and some hepatocytes close to the fibrotic area expressed high levels of TGF-β1, which can induce apoptosis in hepatocytes and stimulate ECM deposition in hepatic fibrosis [44]. We infer that one potential mechanism underlying the protecting effect of DAPT may involve the suppression of TGF-β1 expression, which contributes to hepatocyte proliferation and protects hepatocytes from apoptosis.

Inhibiting γ-secretase can prevent the cleavage of the Notch receptor, blocking Notch signal transduction [30], [31]. The clinical trials with γ-secretase inhibitors have revealed several adverse events, such as gastrointestinal toxicity [45], [46]. Other approaches that target the Notch signaling were recently evaluated and showed promising effects accompanied by a lack of intestinal toxicity in preclinical models [47], [48]. These studies shed light on the clinical implications of γ-secretase inhibitor.

In summary, the present investigation indicates that the Notch signaling pathways become activated in a rat model of liver fibrosis induced by CCl_4_ and that inhibition of Notch signaling exerts potent anti-fibrotic effects in preclinical models. Our study provides the first evidence for the striking suppressive effects of DAPT on hepatic fibrosis. These findings suggest that inhibition of Notch signaling might be a novel option for hepatic fibrosis therapy.

## Materials and Methods

See Materials and Methods S1 for detailed experimental materials and methods.

### Ethics Statement

All the animal related procedures were performed according to the ethical guidelines of the Animal Care and Use Committee of Huazhong University of Science and Technology. Permit numbers: 2011-237. This study was approved by ethics committee of Huazhong University of Science and Technology.

### Reagents and Antibodies

*N*-[*N*-(3, 5-difluorophenacetyl)-l-alanyl]-*S*- phenylglycine *t*-butyl ester (DAPT), a highly active γ-secretase inhibitor, and carbon tetrachloride (CCl_4_) were purchased from Sigma-Aldrich Corporation (U.S.). Lipofectamine™ 2000 transfection reagent was obtained from Invitrogen (Carlsbad, CA, U.S.). The antibodies used for Western blot analysis and immunohistological staining are listed in Table S1.

### Quantitative Real-time PCR Analysis

Total RNA from each sample was extracted using Trizol Reagent (Invitrogen, U.S.) and was reverse-transcribed using PrimeScript RT Master Mix Kit (Takara, Japan) according to the manufacturer’s instructions. Primers for these transcripts are listed in Table S2.

### Western Blotting Analysis

Western blotting analysis was performed according to the manufacturer’s recommended method (Bio-Rad Laboratories, Hercules, CA, U.S.).

### Treatment of Hepatic Fibrosis in Rats

Male Sprague–Dawley rats (180–220 g) were purchased from the Experimental animal center of Tongji Medical College of Huazhong University of Science and Technology (Wuhan, China). Forty male SD rats were randomly allocated into five groups of 8 rats each: Group A served as normal group. These rats received olive oil (the vehicle for CCl_4,_ 3 mL/kg) twice a week for 8 weeks. Rats in group B (model group) received subcutaneous injections of CCl_4_ (3 mL/kg) dissolved in olive oil (2∶3 ratio) twice a week for 8 weeks to induce liver fibrosis as previously described [49], [50]. Rats in group C received CCl_4_ twice a week for 12 weeks and received antifibrotic treatment with DAPT (10 mg/kg) at 8 weeks by intraperitoneal injection daily for another 4 weeks. Rats in group D were treated with CCl_4_ as in group C and received DAPT (50 mg/kg) at 8 weeks daily for another 4 weeks. Rats in group E served as fibrosis group were given the equivalent volumes of CCl_4_ and DMSO (the vehicle for DAPT). Rats in groups A and B were sacrificed at 4 weeks, 8 weeks, and 12 weeks after the first treatment, and the rest of the rats were killed 12 weeks after the initial treatment.

### Histology and Immunohistochemistry

The paraffin-embedded liver sections were stained with hematoxylin-eosin (HE), Masson’s trichrome and Sirius red staining. Immunohistochemical examinations were performed to detect the expression of snail, vimentin, TGF-β1, and proliferating cell nuclear antigen (PCNA).

### Immunoﬂuorescence Staining

Liver tissues were stained with one or different combinations of the following primary antibodies: Notch3, Jagged1, Hes1, and α-SMA. They were visualized under a laser scanning confocal ﬂuorescence microscope (Nikon, Japan).

### Cell Line and Proliferation Assay

HSC-T6 cells were purchased from Cancer Institute and Hospital, Chinese Academy of Medical Sciences (China). They were cultured in DMEM (Gibco, U.S.). At different points in time after DAPT treatment, the number of viable cells was determined colorimetrically at 450 nm using a Cell Counting Kit-8 (Dojindo, Tokyo, Japan).

### Transfection of siRNA

The siRNAs targeting rat Notch3 and a scrambled siRNA that was used as a negative control (NC) were prepared as previously described [32]. HSC-T6 cells seeded in 6-well plates were transfected with Notch3-siRNA or Notch3-NC by Lipofectamine 2000 following the manufacturer’s instructions.

### Assay of Hydroxyproline Content

Total hepatic hydroxyproline levels were determined in the hydrolysates of liver samples according to the protocol provided with the Hydroxyproline Testing Kit (Jiancheng, Nanjing, China) as previously described [49].

### Statistical Analysis

Results are presented as means of three independent experiments (mean ±SD). Statistical analysis of values was performed using the unpaired Student *t* test, with *P* values <0.05 considered significant.

## Supporting Information

Figure S1 Protein levels of Notch 1 and 2 in fibrotic liver. **A**. Rats treated with olive oil or CCl_4_ were killed. The protein levels of Notch1-ICD and Notch2-ICD were analyzed by Western blot analysis. **B.** Expression was normalized against that of β-actin. *P<0.05 versus normal rats. ^#^ *P*<0.05 versus rats at 8 weeks.
(TIF)

Figure S2 Effects of DAPT on apoptosis in fibrotic liver. **A**. Caspase 3 activity in liver lysates was detected by Western blot analysis of normal, fibrosis, and DAPT-treated rats. **B.** Expression was normalized against that of β-actin. *P<0.05 versus rats in fibrosis group and rats treated with DAPT at 10 mg/kg. 1 indicates normal rats; 2 indicates rats in fibrosis group; 3 indicates rats treated with DAPT (10 mg/kg); 4 indicates rats treated with DAPT (50 mg/kg).
(TIF)

Figure S3 Effects of Notch3 knockdown by siRNA on EMT in HSC-T6 cells. **A**. Expression of snail, vimentin, α-SMA, and Notch3-ICD in HSC-T6 cells treated with Notch3-siRNA or the control siRNA (Notch3-NC) were analyzed by Western blotting. **B.** Expression was normalized against that of β-actin. *P<0.05 versus control group.
(TIF)

Materials and Methods S1. (DOCX)

Table S1 Primary antibodies used for Western blot analysis and immunohistological staining.
(DOC)

Table S2 The Primer sequences for TaqMan real-time qPCR.
(DOC)
