# Supplementary figures and images for: Inhibition of Notch Signaling by a γ-Secretase Inhibitor Attenuates Hepatic Fibrosis in Rats
Source: PLoS One. 2012 Oct 3;7(10):e46512. doi: 10.1371/journal.pone.0046512 (PMC3463607; doi:10.1371/journal.pone.0046512)

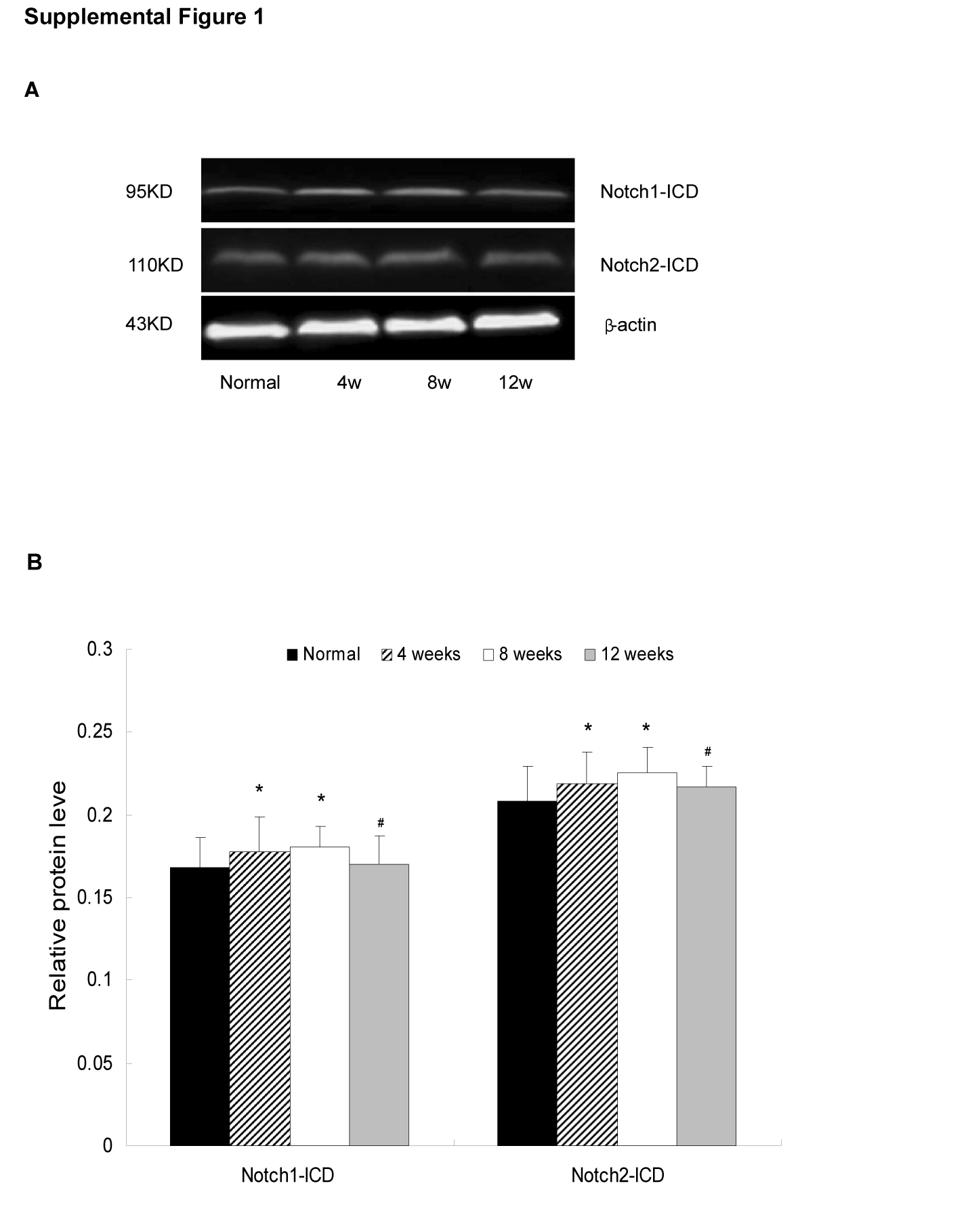

Supplement: Figure S1 — Protein levels of Notch 1 and 2 in fibrotic liver. A. Rats treated with olive oil or CCl4 were killed. The protein levels of Notch1-ICD and Notch2-ICD were analyzed by Western blot analysis. B. Expression was normalized against that of β-actin. *P<0.05 versus normal rats. # P<0.05 versus rats at 8 weeks. (TIF) [file pone.0046512.s001.tif]

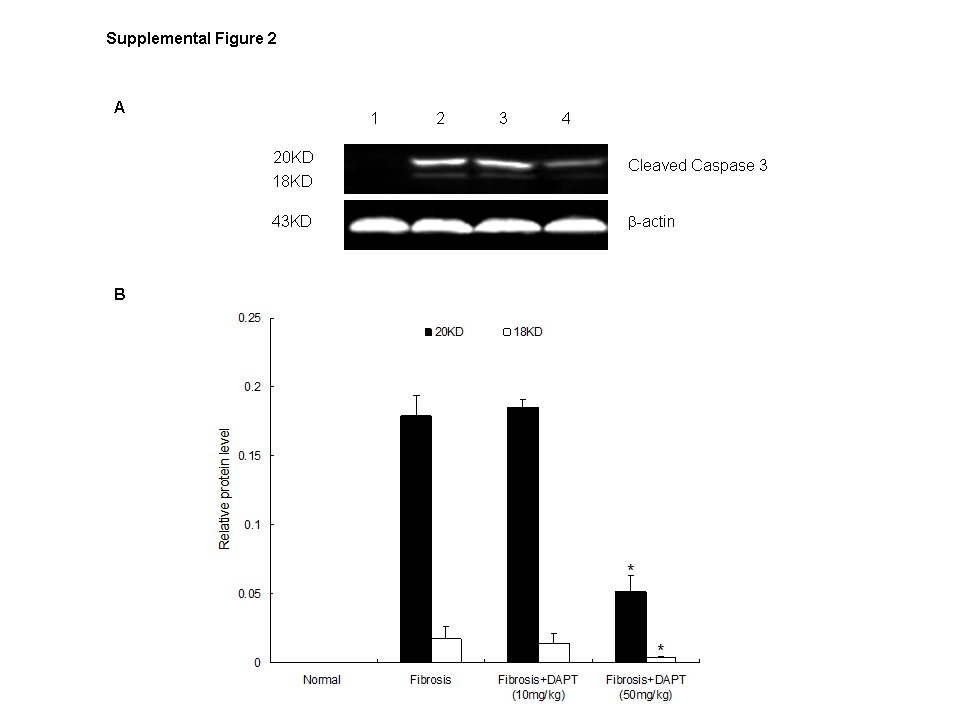

Supplement: Figure S2 — Effects of DAPT on apoptosis in fibrotic liver. A. Caspase 3 activity in liver lysates was detected by Western blot analysis of normal, fibrosis, and DAPT-treated rats. B. Expression was normalized against that of β-actin. *P<0.05 versus rats in fibrosis group and rats treated with DAPT at 10 mg/kg. 1 indicates normal rats; 2 indicates rats in fibrosis group; 3 indicates rats treated with DAPT (10 mg/kg); 4 indicates rats treated with DAPT (50 mg/kg). (TIF) [file pone.0046512.s002.tif]

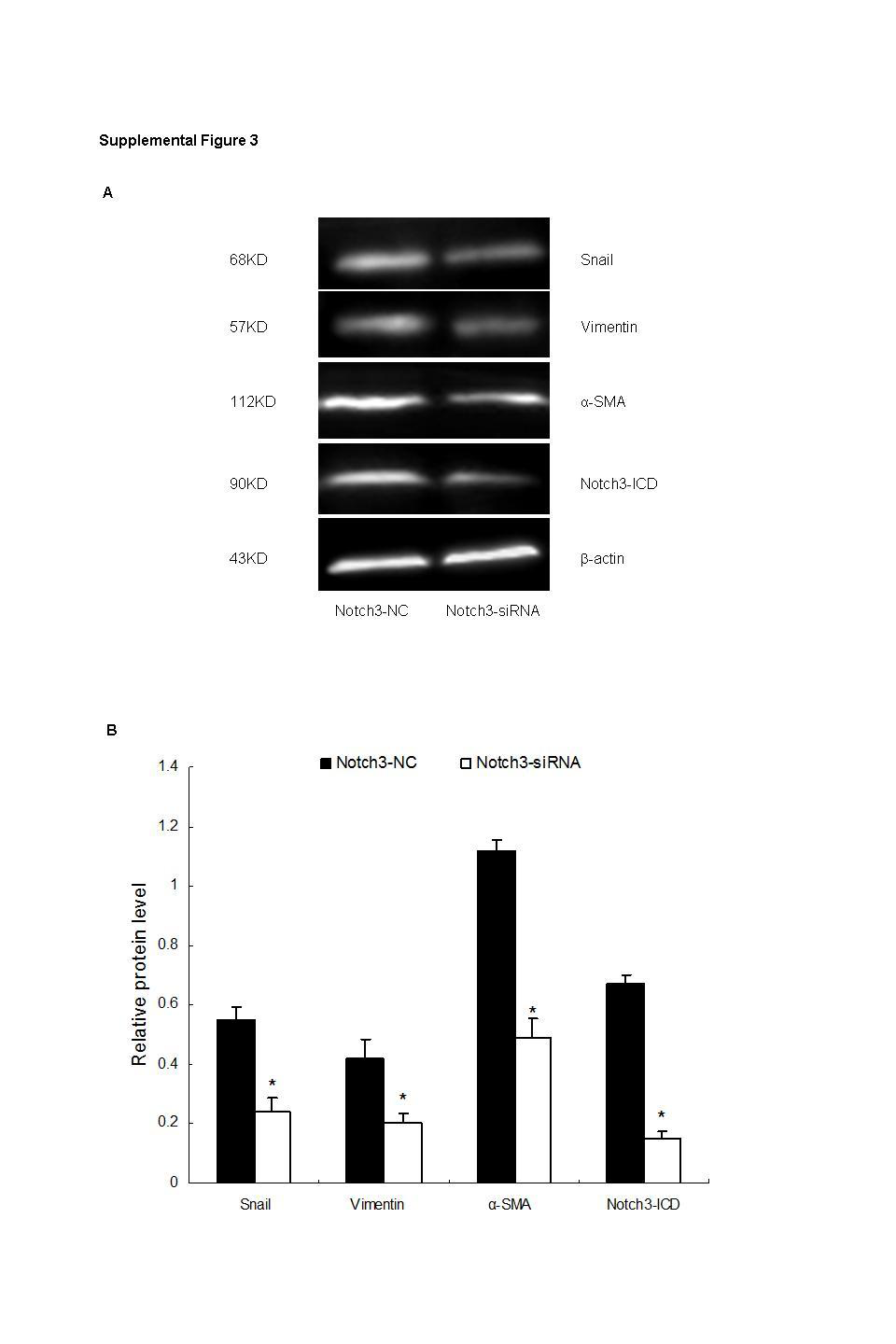

Supplement: Figure S3 — Effects of Notch3 knockdown by siRNA on EMT in HSC-T6 cells. A. Expression of snail, vimentin, α-SMA, and Notch3-ICD in HSC-T6 cells treated with Notch3-siRNA or the control siRNA (Notch3-NC) were analyzed by Western blotting. B. Expression was normalized against that of β-actin. *P<0.05 versus control group. (TIF) [file pone.0046512.s003.tif]
