# Supplementary material for: Inhibition of Notch Signaling by a γ-Secretase Inhibitor Attenuates Hepatic Fibrosis in Rats
Source: PLoS One. 2012 Oct 3;7(10):e46512. doi: 10.1371/journal.pone.0046512 (PMC3463607; doi:10.1371/journal.pone.0046512)
Supplement: Materials and Methods S1. — (DOCX) [file pone.0046512.s004.docx]

**Supplemental Materials and Methods**

**Quantitative real-time PCR analysis**

Total RNA from each sample was extracted using Trizol Reagent (Invitrogen, US) and was reverse-transcribed using PrimeScript RT Master Mix Kit (Takara, Japan) according to the manufacturer’s instructions. TaqMan RT-qPCR was performed using a StepOnePlus (ABI, US) and the Premix Ex Taq(TM) (Takara,Japan). Primer sequences were summarized in Supporting Table 2. The PCR reactions were analyzed using StepOne Software v2.0 (ABI, US). Comparative threshold (Ct) method was used for calculating the relative amount of mRNA of treated sample compared to control samples.

**Histological examination and immunohistochemical staining**

The paraffin embedded liver samples were stained with hematoxylin-eosin (HE), Masson’s trichrome and Sirius red staining. For the semiquantitative analysis of collagen expression, the blue-stained areas in the Masson’s trichrome stained sections were measured on an image analyzer (Image J, NIH) by a technician blinded to the samples. Three fields were selected randomly from each section.

Immunohistochemistry was performed to detect the expression of TGF-β1, EMT-related proteins and proliferating cell nuclear antigen (PCNA). The staining was carried out using StreptAvidin-Biotin Complex (SABC) kit (Boster, China) according to the manufacturer’s instructions. The primary antibodies used for staining are shown in Supporting Table 1.

PCNA-positive hepatocytes (%) were quantiﬁed on image analyzer (Image J, NIH) by a technician blinded to the samples. Three ﬁelds were selected randomly from each section, and a total of 100 hepatocytes were evaluated in every ﬁeld.

**Immunoﬂuorescence staining**

Immunoﬂuorescence was also performed in paraffin-embedded sections with one or with different combinations of antibodies, which are shown in Supporting Table 1. The fluorescent secondary antibodies were conjugated by Dylight488 (goat anti-rabbit, green), Dylight488 (goat anti-mouse, green) or Dylight594 (goat anti-rabbit, red) (Earthox LLC, US). The cell nuclei were counterstained with 4', 6-diamidino-2-phenylindole (DAPI) and the staining was visualized under a laser scanning confocal fluorescent microscope (Nikon, Japan).

Immunoﬂuorescence staining of apoptotic cells was performed by terminal deoxynucleotidyl transferase-mediated deoxyuridine triphosphate nick-end labeling (TUNEL) using the TUNEL Assay Kit (Roche, US). TUNEL-positive hepatocytes (%) were quantified by automated counting performed by image analysis software (ImageJ, NIH) by a technician blinded to the samples. Three fields were selected randomly from each section.

**Western blot assay**

Tissue and cells were lysed in lysis buffer. Protein was quantified by the BCA assay (Beyotime, China), equal amount of protein were separated on 10% SDS-polyacrylamide gels and transferred to polyvinylidene fluoride (PVDF) membrane (Millipore, MA). The membranes were incubated with indicated primary antibody at 4°C overnight followed by horseradish peroxidase-conjugated second antibody for 1 hour at room temperature and detected by chemiluminescence using the BeyoECL Plus (Beyotime, China) with a digital luminescent image analyzer Biospectrum 600 (UVP, US). Quantification of the Western blot data were performed by measuring the intensity of the hybridization signals using ImageJ analysis program. The primary antibodies are shown in Supporting Table 1.

**Cell line and proliferation assay**

HSC-T6 cells purchased from Cancer Institute and Hospital, Chinese Academy of Medical Sciences (China) were cultured in Dulbecco’s-modiﬁed Eagle’s medium (DMEM; Gibco, USA) supplemented with 100 U/mL penicillin, 100 μg/mL streptomycin, and 10% fetal bovine serum (Gibco, USA). At different time point after DAPT treatment, the number of viable cells was determined colorimetrically at 450 nm using the Cell Counting Kit-8 (Dojindo, Tokyo, Japan).

**Transfection of siRNA**

HSC-T6 cells were seeded in 6-well plates at a density of 1×10^5^ cells. The siRNAs were mixed with 5μl Lipofectamine 2000 in 250μl Opti-MEM I medium for 20 min. The transfection mixture was then added to each well with 1.5 ml fetal bovine serum (FBS) free DMEM medium. After 6 h of incubation, the liquid portion was disposed of. Then 2 ml DMEM containing 10% FBS was added and incubated for another 72 h. The following siRNA sequences were used: Notch3 siRNA: 5'-ACAAGAUCAAUACAGGAGCTT-3'; control siRNA sequence: 5'-UUGUACUACACAAAAGUACUG-3'. These siRNA were synthesized by Shanghai Genepharma Co. Ltd. (China).

**Measurement of hepatic hydroxyproline content**

Total hepatic hydroxyproline levels were determined in the hydrolysates of liver samples according to the protocol of Hydroxyproline Testing Kit (Jiancheng, Nanjing, China). The data were expressed as hydroxyproline (μg)/wet liver weight (mg). Three samples were determined randomly from each group.
