# Supplementary material for: Inhibition of Notch Signaling by a γ-Secretase Inhibitor Attenuates Hepatic Fibrosis in Rats
Source: PLoS One. 2012 Oct 3;7(10):e46512. doi: 10.1371/journal.pone.0046512 (PMC3463607; doi:10.1371/journal.pone.0046512)
Supplement: Table S1 — Primary antibodies used for Western blot analysis and immunohistological staining. (DOC) [file pone.0046512.s005.doc]

**Supporting Table 1. Primary antibodies used for Western blot analysis and immunohistological staining**

| **Rat antigens** | **Poly/mono- clonal** | **Manufacturer** | **Dilution** |
| --- | --- | --- | --- |
| α-SMA | monoclonal | Abcam, US | 1:50 (1:300 for Western blot) |
| Jagged1 | monoclonal | Epitomics, Burlingame, US | 1:250(1:5000 for Western blot) |
| Notch1 | polyclonal | Abcam, US | 1:1000 for Western blot |
| Notch2 | polyclonal | Abcam, US | 1:1000 for Western blot |
| Notch3 | polyclonal | Abcam,US | 1:250(1:1000 for Western blot) |
| Hes1 | polyclonal | Santa Cruz Biotechnology, Inc., Santa Cruz, CA | 1:50 (1:100 for Western blot) |
| E-cadherin | polyclonal | Abcam, US | 1:100 |
| Vimentin | monoclonal | Epitomics, Burlingame, US | 1:500(1:1000 for Western blot) |
| Snail | polyclonal | Abcam, US | 1:250(1:500 for Western blot) |
| TGF-β1 | monoclonal | Abcam, US | 1:100(1:1000 for Western blot) |
| PCNA | polyclonal | Santa Cruz Biotechnology, Inc., Santa Cruz, CA | 1:100 |
| Caspase 3 | polyclonal | Abcam, US | 1:1000 for Western blot |
| β-actin | polyclonal | Santa Cruz Biotechnology, Inc., Santa Cruz, CA | 1:1000 for Western blot |
