# Supplementary material for: Inhibition of Notch Signaling by a γ-Secretase Inhibitor Attenuates Hepatic Fibrosis in Rats
Source: PLoS One. 2012 Oct 3;7(10):e46512. doi: 10.1371/journal.pone.0046512 (PMC3463607; doi:10.1371/journal.pone.0046512)
Supplement: Table S2 — The Primer sequences for TaqMan real-time qPCR. (DOC) [file pone.0046512.s006.doc]

Supporting Table 2. The Primer sequences for TaqMan real-time qPCR

| Notch1 | | Forward | | 5'-TTTGCCAACCGGGACATC-3' |
| --- | --- | --- | --- | --- |
|  | | Reverse | | 5'-GTATTCATCCAAAAGCCGCAC-3' |
|  | | Probe | | 5'-ACGGATCACATGGACCGATTGCC-3' |
| Notch2 | | Forward | | 5'-TGCCCAAGGTGTCTTCCAG-3' |
|  | | Reverse | | 5'-GGGTAGTGCCATCATTCATCC-3' |
|  | | Probe | | 5'-TCCGCAACCGAGTAACGGATCTAGAC-3' |
| Notch3 | Forward | | 5'-CCTGCCTGCCTCTATGACAAC-3' | |
|  | Reverse | | 5'-ACACTCCTCGGTGTTACAGCC-3' | |
|  | Probe | | 5'-ACTGCTACTCTGGTGGCCGCGAC-3' | |
| Jagged1 | Forward | | 5'-GTGGAAGAGGATGATATGGATAAGC-3' | |
|  | Reverse | | 5'-CTCCTCTCTGTCTACCAGCGTGTAC-3' | |
|  | Probe | | 5'-CCAGCAGAAAGTCCGGTTTGCCA-3' | |
| Hes1 | Forward | | 5'-TGCTACCCCAGCCAGTGTC-3' | |
|  | Reverse | | 5'-GCTTTGATGACTTTCTGTGCTCA-3' | |
|  | Probe | | 5'-CTGTCTTTGGTTTGTCCGGTGTCGT-3' | |
| GAPDH | Forward | | 5'-gatgacatcaagaaggtggtgaag-3' | |
|  | Reverse | | 5'-accctgttgctgtagccatattc-3' | |
|  | Probe | | 5'-ACTTCAACAGCAACTCCCACTCTTCCACC-3' | |
